# Analysis of the Renal Protection and Antioxidative Stress Effects of Panax notoginseng Saponins in Diabetic Nephropathy Mice

**DOI:** 10.1155/2022/3610935

**Published:** 2022-10-05

**Authors:** Wei Mi, Min Yu, Shuying Yin, Yuhan Ji, Tala Shi, Ning Li

**Affiliations:** ^1^School of Public Health and Management, Binzhou Medical University, Yantai, 264003 Shandong, China; ^2^School of Pharmacy, Binzhou Medical University, Yantai, 264003 Shandong, China; ^3^School of Second Clinical Medicine, Binzhou Medical University, Yantai, 264003 Shandong, China

## Abstract

**Objective:**

Diabetic nephropathy (DN), a diabetes-induced chronic complication, is the major trigger of end-stage renal disease. As the main active ingredient of Panax notoginseng (PNG), Panax notoginseng saponins (PNS) are crucial in treating renal diseases. This study is aimed at investigating the role played by PNS in renal protection and antioxidative stress (OS) in DN mice.

**Methods:**

A DN mouse model was constructed, and then low, medium, and high doses of PNS were used to intervene the model group mice. Eight weeks after intervention, the 24 h urine protein (UPro) and urinary albumin (UAlb) were quantitatively examined, and the related blood biochemical indices were measured. HE and PAS staining were performed for pathological changes of renal tissue. ELISA and western blotting were carried out to quantify the levels of OS indexes and inflammatory factors (IFs) in mouse kidney tissues and the expression of nuclear factor erythroid 2-related factor 2 (Nrf2) and heme oxygenase-1 (HO-1), respectively.

**Results:**

The weight of DN mice decreased first compared with control animals and then gradually increased after different doses of PNS treatment. Besides, DN mice presented elevated urine volume, UPro, and UAlb, all of which were reversed by PNS intervention. SOD activity and GSH content in renal tissues of the model group mice decreased markedly versus the control group, and MDA, CRP, IL-6, and TGF-*β*1 contents elevated statistically, while different doses of PNS effectively reduced the OS injury and IFs in mice. Compared with the model group, PNS dose-dependently increased Nrf2 and HO-1 levels in DN mice.

**Conclusions:**

PNS is protective of HFF + STZ-induced DN mice against kidney tissue damage and can reduce the excretion of UPro and relieve the OS state of mice, possibly by activating Nrf2/HO-1 axis to play an antioxidant and anti-inflammatory role.

## 1. Introduction

As the most prevalent and serious consequence of diabetes mellitus (DM), diabetic nephropathy (DN) is related to elevated morbidity and mortality in DM patients [1, 2]. DM shows a rapid growth in prevalence globally, particularly in developing countries [3]. The past decade has also witnessed a sharp rise in the incidence and prevalence of DN in China, with an estimated 24.3 million Chinese patients with DM and chronic renal disease [4] and a tenfold risk of developing end-stage renal failure (ESRF) in DM patients [5]. The International Diabetes Federation (IDF) reported a possibility of 40% for developing ESRF in diabetic patients, and DM and hypertension (HT), either combined or alone, were found in approximately 80% among all ESRF cases [6]. Glomerular basement membrane (GBM) thickening, albuminuria, and normal glomerular filtration rate (GFR) are the characteristics of DN at the initial stage; HT is always present within 5 years since GBM thickening, followed by mild to severe mesangial dilation [7]. Modern treatment protocols and promising new therapies are essential for DN.

The two most prominent risk factors of DN are hyperglycemia and HT [8]. Western medicines such as renin-angiotensin system (RAS) inhibitors are the main drugs to treat DN [9]. In a randomized controlled trial, it was found that, besides drugs for controlling blood glucose (BG) and blood pressure, only RAS inhibitor therapy demonstrated a strong renal protective effect [10]. However, there is an unmet need for treatment in patients who are intolerant or unresponsive to current clinical drug therapy, as well as in patients with deteriorating renal function and normal albuminuria [11, 12]. Recently, growing evidence has confirmed the exact efficacy of traditional Chinese medicine (TCM) for DN. Plants were widely used for medical purposes long before recorded history [13]. TCM theory indicates that the main nosogenesis of DN is the kidney, with kidney deficiency and kidney collateral stagnation as the key to DN [14, 15], and Yin asthenia generating intrinsic heat and Qi-blood stasis as the major triggers, while the effective active components of TCM have been indicated by modern studies to enhance peripheral blood supply and achieve the purpose of treatment [16, 17]. Screening of promising drug candidates from natural products, including TCMs for the relief of DN-related symptoms, may shed some light on the promotion of new therapeutic drugs and strategies for DN patients [18].

Panax notoginseng (PNG), also known as pseudoginseng in China [19], is a highly valued and extensively used Chinese herb in Asia, where it has been used to treat cardiovascular diseases and DM for thousands of years [20]. Notoginsenoside is the main active component of PNG, with some studies indicating that Panax notoginseng saponins (PNS) can reduce BG and blood lipid levels [21, 22]. Recent research has shown that Panax notoginseng saponin R1 plays a protective role in DN through Nrf2 pathway [23] while effectively delaying the process of renal interstitial fibrosis and protecting the kidney from injury [24]. Besides, PNS is protective of the kidney by inhibiting oxidative stress (OS) and reducing cisplatin-induced nephrotoxicity [25]. However, the improvement and mechanism of PNS on DN-induced renal pathological damage caused have not been reported. Consequently, this study revealed the related mechanism of PNS on renal protection of DN mice from the level of OS injury and inflammation.

## 2. Data and Methods

### 2.1. Experimental Animals and Model Building

Male C57BL/6 mice of specific-pathogen-free (SPF) grade, with the age of 6-8 weeks and the weight of 20-25 g, were supplied by Shanghai Animal Experimental Center. They were caged according to the requirements for SPF animals, and the control environment (temperature: 22 ± 2°C, humidity: 55% ± 2%) was in a ventilated and quiet state. Mice could drink and eat freely and were given adaptive feeding for 7 days, after which group experiments were carried out.

The animals were stochastically assigned to control (*n* = 9) and model (*n* = 41) groups, of which the latter was treated with DN modeling. Mice were given high-fat feeding (HFF: 65.0% common diet, 20% sucrose, 10% lard, and 5% cholesterol). Six weeks later, intraperitoneal streptozotocin (STZ, dissolved in 0.1 mmol/L citric acid buffer, pH 4.4, 30 mg/kg) was given for five consecutive days to establish a DN mouse model. Control group animals were fed normally and injected with the same amount of citrate buffer into the abdominal cavity. If the BG content of mice after feeding for 7 days was measured to be higher than 16.7 mmol/L with the urinary protein (Upro) increased by more than ten times versus the control mice, the model was considered as successful. The Institutional Animal Care and Use Committee has given their approval for all the animal experiments.

### 2.2. Grouping and Administration

36 mice were successfully modeled and randomized into four groups with 9 mice in each group: model, as well as low- (L-PNS), medium- (M-PNS), and high-dose (H-PNS) groups of PNS. On the second day after successful modeling, mice in L-, M-, and H-PNS groups were intragastrically administrated with 50, 100, and 200 mg/kg of PNS (Sanjiu Medical & Pharmaceutical), respectively, while control and model mice were given normal saline of equal volume, lasting for 8 weeks. All the animals were kept in separate cages throughout the experiment, during which they drank and ate freely, and did not inject insulin or other drugs that could lower BG. There was no mouse death at the end of the experiment.

### 2.3. Biochemical Index Detection and Specimen Collection

After 8 weeks of drug administration, the mice were weighed and put into a metabolic cage to collect 24 h urine to measure urine volume. UPro was quantitatively evaluated by UPro quantitative test kits (CBB method, Nanjing Jiancheng Institute of Bioengineering, China). ELISA measured urine albumin (UAlb) content following the instructions of the kit (Nanjing Jiancheng Bioengineering Institute, China). BG level was determined via tail vein blood sampling. Subsequently, blood was collected from the abdominal aorta of the anesthetized animals, to quantify concentrations of serum creatinine (Scr) and blood urea nitrogen (BUN) with an automatic biochemical analyzer. The bilateral kidneys of mice were excised, some of which were treated with 4% paraformaldehyde, followed by routine paraffin-embedding and slicing; the other part was refrigerated (-80°C) for later use. All tests were carried out uniformly after the collection of all samples.

### 2.4. Histological Staining

We observed the pathological changes of kidney tissue with an optical microscope after HE and PAS staining of paraffin sections. Image-Pro Plus 5.1 from Media Cybernetics Inc., Bethesda, MD, quantified glomerular area and mesangial matrix expansion.

### 2.5. Detection of OS and Inflammatory Factors (IFs)

After kidney tissue collection from each group, the tissues were treated with cleaning with normal saline, blood removal, homogenation on ice bath, and 10 min of centrifugation (4°C, 10000 r/min) to obtain supernatant for quantitative determination. According to the instructions of the corresponding kit, kidney tissue superoxide dismutase (SOD), malondialdehyde (MDA), glutathione (GSH), C-reactive protein (CRP), transforming growth factor (TGF)-*β*1, and interleukin (IL)-6 were determined. All kits were purchased from Nanjing Jiancheng Bioengineering Institute.

### 2.6. Western Blotting Analysis

A protein extraction kit (Beyotime, China) isolated total protein in kidney tissue, and protein concentration was detected by the BCA method. The prepared SDS-PAGE gel was placed in an electrophoresis tank, and the protein was mixed evenly after being boiled in a water pot for 5 min. Then, maker and sample protein, 20 *μ*L each, were added into the wells and moved to a PVDF membrane to seal with 5% nonfat dried milk. After rinsing, the membrane immersed in corresponding primary antibodies all from Abcam, namely, Nrf2 (1 : 1,000), HO-1 (1 : 2,000) and *β*-actin (1 : 1,000), for overnight culture (4°C). Following TBST rinsing, it was immersed in diluted horseradish peroxidase (HRP) labeled goat anti-rabbit IgG secondary antibody (1 : 2,000, Abcam) for 60 min of cultivation on a horizontal shaker. The membrane, after being washed by TBST, was developed by ECL, and the protein bands were analyzed by ImageJ software (NIH ImageJ; NIH, Bethesda, MD) and normalized using *β*-actin.

### 2.7. Statistics and Analysis

We performed data analyses using SPSS 20.0 (Chicago, IL, USA) and GraphPad Prism 8.0 (GraphPad, San Diego, CA). Each test was independently conducted three times, with the average values represented by mean ± standard deviation. After Student's *t*-test or one-way ANOVA, data were further analyzed by Bonferroni post hoc testing, with *P* < 0.05 indicating the presence of significance.

## 3. Results

### 3.1. Impacts of PNS on Symptoms of DN Mice

**Figure 1:**
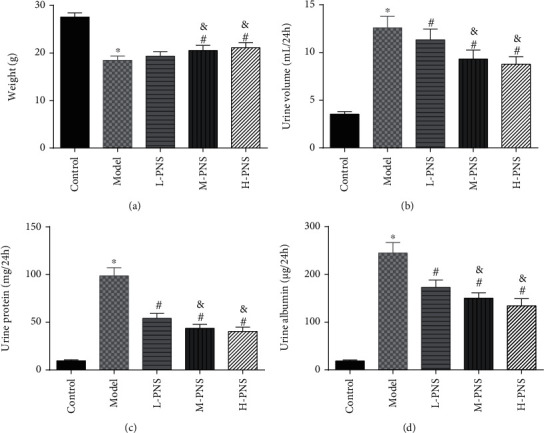
Symptoms of diabetic mice ((a) weight change of mice. (b) 24 h urine volume of mice. (c) 24 h urine protein content of mice. (d) 24 h urine albumin of mice;^∗^*P* < 0.05 vs. control; ^#^*P* < 0.05 vs. model; ^&^*P* < 0.05 vs. L-PNS).

We first investigated the impact of PNS on HFF + STZ injection-induced DN mice. As shown in Figure 1, the weight of DN mice decreased at first and then gradually increased after treatment with different doses of PNS (*P* < 0.05, Figure 1(a)). In addition, DN mice showed increases in 24 h urine volume, UPro, and UAlb than control animals; however, PNS treatment reversed these results, with the above parameters in the M- and H-PNS group being lower than the L-PNS group (*P* < 0.05, Figures 1(b)–1(d)).

### 3.2. Impacts of PNS on Biochemical Indices of DN Mice

**Figure 2:**
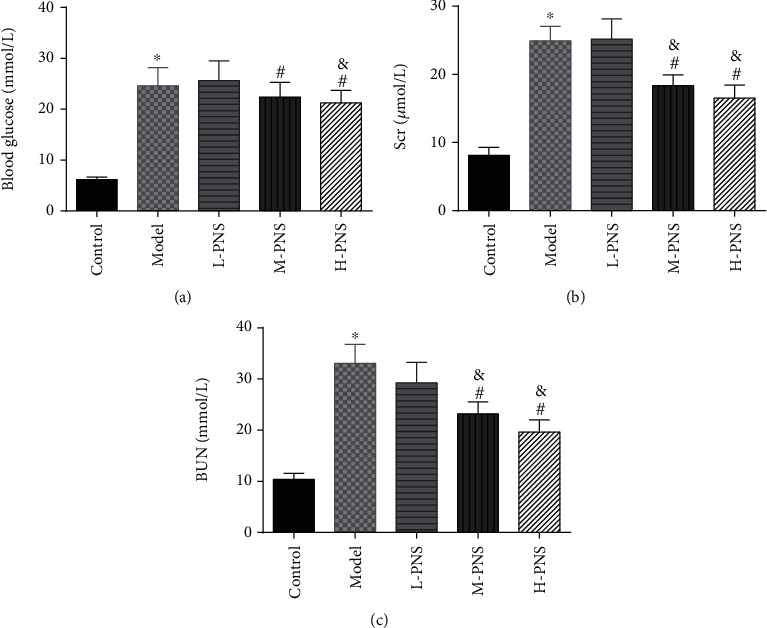
Detection of blood biochemical indices in mice ((a) blood glucose level. (b) Blood creatinine level. (c) Blood urea nitrogen level; ^∗^*P* < 0.05 vs. control; ^#^*P* < 0.05 vs. model; ^&^*P* < 0.05 vs. L-PNS).

As presented in Figure 2, the model group had higher BG, Scr, and BUN levels than the control group (*P* < 0.05), while the BG, Scr, and BUN in M- and H-PNS groups decreased remarkably versus model group animals (*P* < 0.05). It suggests that PNS can lower BG level and enhance renal function in DN mice.

### 3.3. Renal Histopathological Changes in Mice

**Figure 3:**
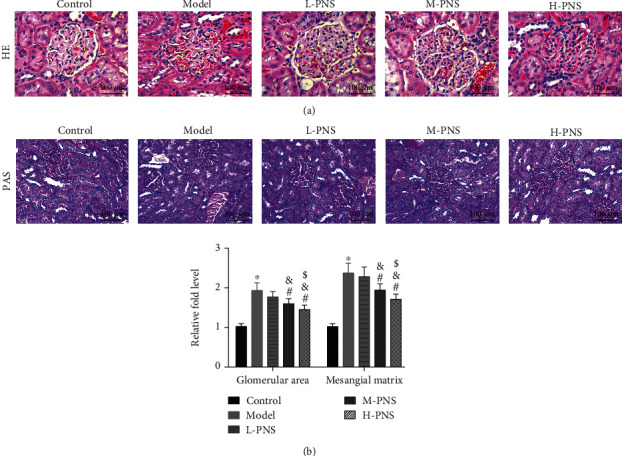
Renal histopathological changes in mice ((a) HE staining. (b) PAS staining, glomerular area, and mesangial matrix expansion of mice; original magnification: ×400; ^∗^*P* < 0.05 vs. control; ^#^*P* < 0.05 vs. model; ^&^*P* < 0.05 vs. L-PNS; ^$^*P* < 0.05 vs. M-PNS).

HE staining of renal tissues of mice in each group (Figure 3(a)) showed that the glomeruli of the control group mice were normal in size and morphology, with regular arrangement of renal tubules and clear structure. In the model group, the kidney damage occurred, the glomerular volume was normal and irregular, and the renal tubules were disordered, while each PNS treatment group showed relieved kidney damage than model group. With the increase of dosage, the glomerular morphology and size tended to be normal, and the arrangement of renal tubules tended to be regular.

PAS staining of renal tissues of mice in each group (Figure 3(b)) revealed that the glomeruli of control mice were normal in volume and complete in structure. In the model group, kidney damage, irregular glomerular volume, and mesangial matrix deposition occurred. While the renal injury was alleviated in each PNS administration group, and with the increase of dosage, the glomerulus morphology and size tended to be normal, the mesangial matrix deposition degree reduced, and the glomerular area and mesangial matrix expansion decreased dose-dependently (*P* < 0.05). The results suggest the ability of PNS to alleviate renal tissue damage in DN mice.

### 3.4. Impacts of PNS on OS Index in DN Mice

**Figure 4:**
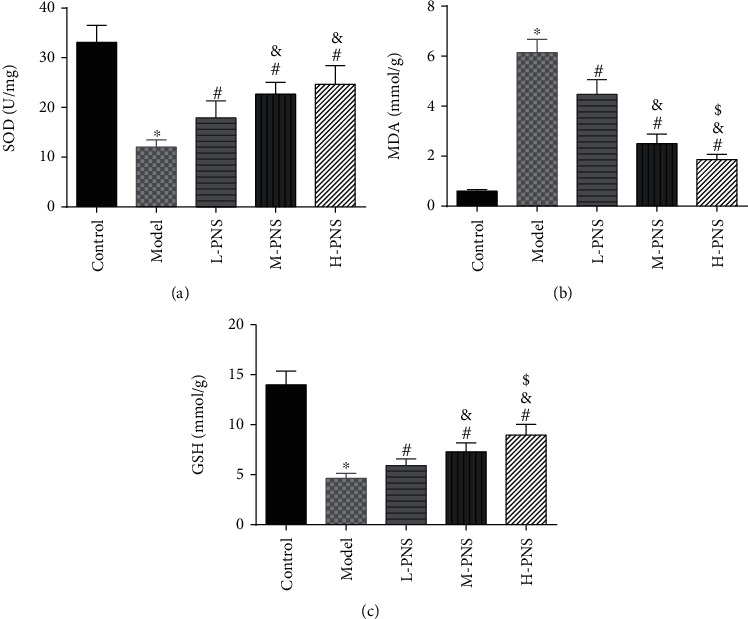
Changes of oxidative stress indicators ((a) SOD activity. (b) MDA content. (c) GSH content; ^∗^*P* < 0.05 vs. control; ^#^*P* < 0.05 vs. model; ^&^*P* < 0.05 vs. L-PNS; ^$^*P* < 0.05 vs. M-PNS).

As shown in Figure 4, SOD activity and GSH content in the model group decreased statistically and MDA increased, versus the control group (*P* < 0.05). In comparison with the model group, the differences of the above indexes in L-, M-, and H-PNS groups were statistically significant, and the contents of MDA and GSH were dose-dependent (*P* < 0.05). It can be seen that PNS can enhance the anti-OS capacity of renal tissue in DN mice and reduce the OS resultant kidney injury.

### 3.5. Impacts of PNS on IFs in Renal Tissue of DN Mice

**Figure 5:**
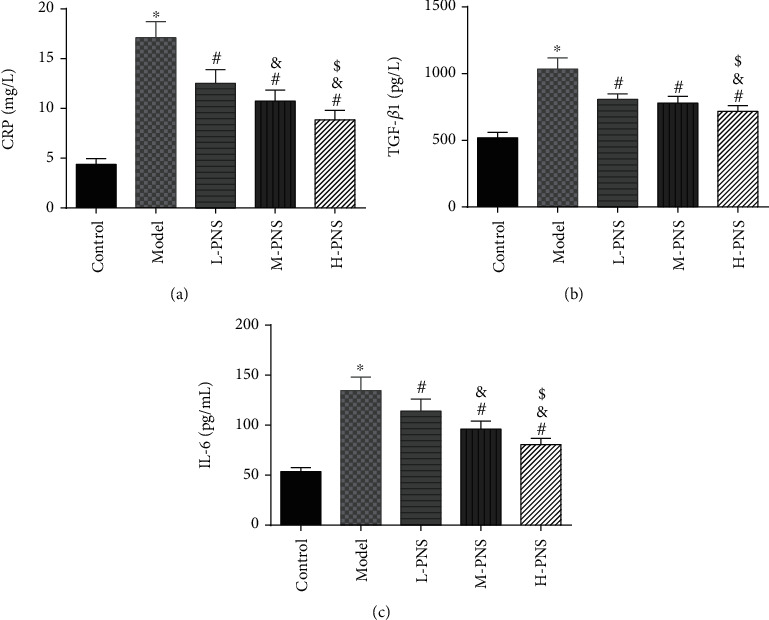
Changes of inflammatory factors in renal tissue ((a) CRP level. (b) TGF-*β* 1 level. (c) IL-6 level; ^∗^*P* < 0.05 vs. control; ^#^*P* < 0.05 vs. model; ^&^*P* < 0.05 vs. L-PNS; ^$^*P* < 0.05 vs. M-PNS).

As shown in Figure 5, CRP, kidney tissue IL-6, and TGF-*β*1 concentrations were evidently higher in the model group mice versus control mice (*P* < 0.05), while L-, M-, and H-PNS groups showed reduced contents of CRP, IL-6, and TGF-*β*1 in kidney tissue than the model group. Among the three PNS administered groups, the M-PNS group had lower CRP and IL-6 contents than the L-PNS group, and the H-PNS group exhibited lower CRP, IL-6, and TGF-*β*1 than the M-PNS group, all with statistical significance (*P* < 0.05).

### 3.6. Nrf2 And HO-1 Protein Levels in Mouse Kidney

**Figure 6:**
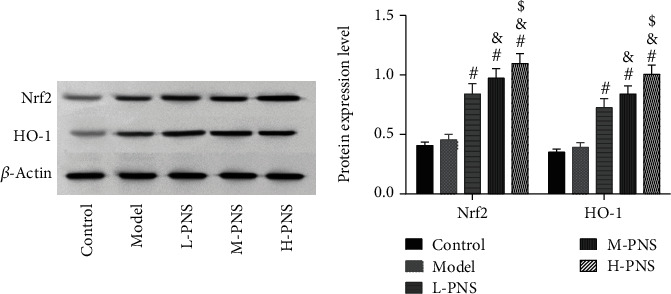
Nrf2 and HO-1 protein expression in kidney tissue (^∗^*P* < 0.05 vs. control; ^#^*P* < 0.05 vs. model; ^&^*P* < 0.05 vs L-PNS; ^$^*P* < 0.05 vs. M-PNS).

As shown by western blotting in Figure 6, the model group mice had increased but not statistically significant Nrf2 and HO-1 in kidney tissue compared with control mice (*P* > 0.05). And PNS dose-dependently increased Nrf2 and HO-1 expression compared with the model group mice (*P* < 0.05).

## 4. Discussion

DN, a kind of diabetic glomerulosclerosis with known inducements such as genetic factors, is a metabolic disorder of the glomeruli accompanied by vascular damage and changes in glomerular hemodynamics [26]. Referring to past literature, we used HFF + STZ injection, a widely used method to simulate DN in vivo [27, 28], to induce DN in mice in this study. After 8 weeks of administration, the contents of 24 h Upro, 24 h UAlb, and Scr in mice increased, and the mesangial matrix increased significantly. Early DN is characterized by morphological and ultrastructural changes, including GBM thickening, glomerular hypertrophy, and tubular atrophy. Unsurprisingly, we observed glomerular hypertrophy and disordered renal tubules in mice after HE staining and PAS staining of mouse kidney tissues. The above experimental results indicated the success construction of an animal DN model in this study.

PNS is an effective active ingredient extracted from PNG, which has the functions of blood vessel dilation, enhancing microcirculation, anti-inflammation, anti-oxidation, anti-shock, etc., with good activities in cardio-cerebrovascular systems, as well as anti-inflammation and antitumor actions [29]. PNS can protect the early kidney of type 1 DM rats via suppressing VEGF protein expression and elevating BMP-7 in the kidneys [30]. Another report showed that PNs protect the kidney by inhibiting TGF-*β*1 expression and enhanced Smad7 expression [31]. In this study, different doses of PNS were administered to the modeled mice. The results revealed the ability of PNS to alleviate glomerular injury, with basically recovered glomerular size and morphology in M- and H-PNS groups, and relatively normal arrangement of renal tubules, indicating that PNS could protect glomerular structure from major changes. DN progresses through early renal decline, middle-stage marked proteinuria, and finally to end-stage renal disease. The change from normal renal function indexes to altered ones, that is, the continuous increase of Scr and BUN, is considered to be the initial state of DN [32]. Creatinine is hardly absorbed by renal tubules but is filtered out by the glomerulus, with its abnormally high levels in the blood indicating a reduced GFR [33]. High levels of BUN suggest decreased tubule reabsorption and decreased glomerular filtration [34]. After PNS intervention, the levels of Scr and BUN in the model group mice decreased, suggesting the protection of PNS against DN. It is generally believed that OS is critical in the process of diabetic kidney injury [35, 36]. Persistent hyperglycemia can give rise to the abnormality of oxidative free radicals in tissues and cells, while reactive oxygen species (ROS), as intracellular OS signal molecules, mediate the occurrence and development of renal tissue damage in DN. ROS stimulates lipids to cause lipid peroxidation (LPO), which induces the increase of MDA—the final decomposition product of LPO whose level can reflect LPO rate or intensity in tissues and cells [37]. In the antioxidant enzyme system, SOD is essential in the oxidation and antioxidant balance of the body, catalyzing superoxide anion in cells into relatively stable hydrogen peroxide, which turns into H_2_O under the action of catalase and glutathione peroxidase (GSH) to protect tissues and cells from damage [38]. There is obvious OS reaction in kidney tissue of DN mice that leads to the decrease of renal function, and the oxidation-antioxidation imbalance can aggravate the occurrence and development of DN. This study found decreased kidney tissue GSH and SOD contents and increased MDA in the model group mice compared with control animals, indicating decreased antioxidant capacity of kidney tissue of the model group mice. And in comparison with the model group, M- and H-PNS groups exhibited elevated GSH and SOD and decreased MDA, which suggests that PNS can alleviate the OS injury of kidney caused by DM and improve the body's antioxidant capacity. We also found increased expression of Nrf2/HO-1 pathway-related proteins after PNS administration and stimulated HO-1 levels. Nrf2 signaling pathway is an extremely important endogenous antioxidant stress pathway discovered so far. Nrf2 translocation to the nucleus regulates transcription and can activate the transcription of multiple antioxidant genes and phase II detoxification enzyme genes (HO-1, SOD, GSH, etc.), thus scavenging the accumulated excessive ROS and improving the ability of cells to resist OS [39, 40].

In addition, OS and immune inflammation are often associated with the development of DM and DN [41]. The occurrence and development of DN are often accompanied by elevated levels of acute phase inflammatory markers (e.g., CRP and IL-6), suggesting the involvement of inflammation in diabetic kidney injury. IL-6, a multifunctional cytokine that is crucial in the acute phase reaction and the synthesis of CRP by hepatocytes, is also a major circulating substance in the body linking systemic immune responses with local vascular injuries. Under pathological conditions, IL-6 can promote T and B cell overactivation and expansion, accelerate cell apoptosis, and promote islet B cell destruction and renal mesangial proliferation [42]. IL-6 also induces CRP, which in turn promotes monocyte adhesion to endotheliocytes, directly damaging vascular endotheliocytes and aggravating the vascular-damaging effects of other IFs [43]. With the ability to cause renal fibrosis, TGF-*β*1 is an important influencing factor that has attracted the attention of domestic and foreign scholars due to its role in DN [44]. TGF-*β*1 is shown to induce the chemotaxis of inflammatory cells and monocytes to synthesize cytokines like IL-6 and improve their biological activity [45]. Studies have demonstrated that PNS inhibited inflammation via reducing the induction of inflammatory cytokines and TGF-*β*1 [46]. Moreover, antioxidant proteins in rats could be activated by PNS [24]. Our findings demonstrated significantly increased IL-6, CRP, and TGF-*β*1 in DN mice, which was statistically reduced by PNS, suggesting the capacity of PNS to effectively reduce inflammation in kidney tissue of DN mice.

## 5. Conclusion

Taken together, PNS is protective of HFF + STZ-induced DN mice, further confirming that PNS may have protective effects on mouse kidney through antioxidant and anti-inflammatory actions, possibly through the Nrf2/HO-1 pathway.

## Data Availability

The labeled dataset used to support the findings of this study are available from the corresponding author upon request.
